# Phylogenetic screen for important RNA structure motifs in the HIV-1 genome

**DOI:** 10.1186/1742-4690-8-S2-P40

**Published:** 2011-10-03

**Authors:** Stefanie A Knoepfel, Ben Berkhout

**Affiliations:** 1 Laboratory of Experimental Virology, Department of Medical Microbiology, Center for Infection and Immunity Amsterdam (CINIMA), Academic Medical Centre, University of Amsterdam, Meibergdreef 15, 1105 AZ Amsterdam, The Netherlands

## Background

Several HIV-1 RNA structures play important roles in viral replication. We showed that despite sequence divergence among virus isolates, certain structural motifs such as the TAR hairpin are conserved [1]. The structure of the complete 9kb HIV-1 RNA genome was determined via SHAPE-technology in 2009, which revealed many new structural motifs [2]. We speculate that important RNA structures are conserved despite sequence variation, and we used the SHAPE-derived RNA structure model to screen for new structured RNA signals.

## Materials and methods

Sixteen hairpin motifs scattered along the HIV-1 RNA genome were selected and evaluated in silico for structural conservedness. As reference sequence we used the HIV-1 NL4-3 strain that was analyzed by SHAPE [2]. The most prevalent sequences for HIV-1 subtypes A, B, C, and D, as well as SIVchimp in the Los Alamos database were chosen as the 'prototype'. This prototype sequence was analyzed with the mfold program and the predicted structure was compared to that of the NL4-3 reference. Four hairpin motifs revealed only minor structural variations in subtypes A-D and SIVchimp and were analyzed in further detail using the HIV sequence compendium 2010 with up to 34 sequences per subtype. All sequence variations were evaluated for maintenance of the structure. To investigate the impact of the 4 hairpin motifs on HIV-1 replication, silent codon changes were introduced into the full length molecular clone HIV-1 LAI, either per individual hairpin or all 4 combined.

## Results

Only 4 of the 16 HIV-1 RNA structures are well-conserved in the HIV-1 subtypes and SIVchimp. Three hairpin candidates are positioned within the reverse transcriptase gene, one is located in the Nef/3'LTR overlap. Based on the HIV-1/SIV full length sequences in the HIV sequence compendium 2010, we could show that the majority of reported sequence variations maintained the RNA structure. We introduced silent codon changes to destabilize the RNA hairpins for each of the 4 motifs, which was confirmed in silico by mfold predictions. Pilot cell culture experiments indicate that the mutation of individual RNA motifs has little impact on the virus replication rate, but the combined structure alterations in 4 regions affect the HIV-1 fitness.

## Conclusions

It is likely that many yet undisclosed structured RNA signals reside in the HIV-1 RNA genome. Our strategy is to first map the RNA structures that are phylogenetically conserved among the HIV/SIV isolates, followed by a mutational test of the respective structures. Of the important motifs, follow-up studies should reveal the role played by the RNA signal in HIV-1 replication.
